# Metabolite labelling as a tool to define hierarchies in *Clostridium acetobutylicum* sugar usage and its relevance for biofuel production

**DOI:** 10.1111/1751-7915.12696

**Published:** 2017-02-21

**Authors:** María Hidalgo, Elena Puerta‐Fernández

**Affiliations:** ^1^ Departamento de GenéticaUniversidad de SevillaAvda. Reina Mercedes, 641012SevillaSpain

## Abstract

This is a highlight on article ‘Metabolite labelling reveals hierarchies in *Clostridium acetobutylicum* that selectively channel carbons from sugar mixtures towards biofuel precursors’ by Ludmilla Aristilde.

The finite nature of fossil‐based resources, as well as the environmental awareness of the effects of their exploitation, has led to an increasing interest in finding alternative ways to produce chemicals, materials and energy. In terms of energy, biofuels are a good option for decreasing the environmental effects caused by fossil fuels, and in the last years, a great effort has been made to find industrial feedstocks for their production that do not compete with human feeding. Agricultural lignocellulosic waste seems as a good alternative for the currently used feedstock (mainly corn), and several companies are investing great efforts in developing efficient pre‐treatment of the material, as well as appropriate enzymatic cocktails, to ensure the release of monomeric sugars from this material.

Although bioethanol is the most known and industrially produced biofuel, biobutanol is also a good alternative, showing, compared to ethanol, a higher energy content and lower volatility, being also less corrosive (Jin *et al*., 2011). A well‐known route for the biological production of butanol is the clostridial ABE route. ABE stands for acetone, butanol and ethanol and defines a pathway shared for several *Clostridium* species, known as solventogenic Clostridia, that convert sugars into these three solvents during their growth (Dürre, 1998). The ABE fermentation was industrially exploited in Europe and USA during World War I, to obtain acetone and to produce cordite, a propellant compound utilized in munition. At that time, butanol was a worthless co‐product, although it raised in value just after the war, being used by the nitrocellulose lacquer industry (Gabriel, 1928). These industrial processes ended in 2004 due to economic reasons (Schiel‐Bengelsdorf *et al*., 2013). Interestingly, in the last years, there is a rising interest in butanol fermentation, due to, in the one hand, the increasing interest in biofuels versus fossil fuels, and, in the other hand, the instability of the ethanol market, and the possibility of using butanol, not only as a fuel, but also as a bulk chemical.

Several solventogenic Clostridia have been studied for their butanol production. The most widely studied strain is *Clostridium acetobutylicum*, followed by *Clostridium beijerinckii*. Clostridial strains are good candidates for fermenting lignocellulosic material, because they belong to the number of anaerobic bacteria that naturally contribute to the decomposition of lignocellulosic wastes in soils. In terms of butanol production, several mutants have been developed, both by random mutagenesis or engineered, that increase the butanol production of their parental strains. Usually, the butanol hyperproducing phenotype is obtained because the strain shows a higher tolerance to the solvent (butanol, Lin and Blaschek, 1983; Liu *et al*., 2013). Another strategy to increase butanol production relies on directing the metabolism of the bacteria to decrease the production of the other solvents, changing the normal 3:6:1 acetone:butanol:ethanol ratio, to a higher butanol ratio, either avoiding acid formation (Jang *et al*., 2012) or increasing their re‐assimilation (Xu *et al*., 2014). Also, when fermenting a complex substrate, such as lignocellulosic wastes, the efficient utilization of all fermentable sugars is important, and some of the hyperproducing strains described show a better assimilation of C5 sugars (Ren *et al*., 2010).

The recent work by Ludmilla Aristilde (Aristilde, 2017) uses metabolic labelling to elucidate the fate of the carbons from the different sugars present in a lignocellulosic sample, after fermentation by *Clostridium acetobutylicum*. She applies an already established method (high‐performance LC‐MS) to identify the metabolites that can clarify whether the carbons are being used for biofuels synthesis. The main sugars present in lignocellulosic samples are C6 sugars (glucose) from the cellulose and both C6 and C5 sugars from the hemicellulose. The hexoses in hemicellulose are galactose and mannose, whereas the pentoses are xylose and arabinose. However, it is worth noting that the actual composition of lignocellulosic materials varies widely depending on the origin of the material, as well as the previous treatment to which the material is subjected to ensure sugar release (Chandra *et al*., 2007). In terms of sugar utilization, it has already been described that *C. acetobutylicum* can use any of these sugars when fed as sole carbon source and that glucose usage is preferential in complex sugar mixtures (Aristilde, 2017). In this study, published in this issue, through metabolite labelling, the author has demonstrated that, when in a mixture with glucose, galactose is not assimilated by *Clostridium acetobutylicum*, whereas mannose can be assimilated. When determining the fate of the hemicellulosic hexoses, galactose is not routed towards butyril‐coA (although, surprisingly, there is a higher biosynthetic flux of butyryl‐CoA from feeding on the glucose:galactose mixture than from feeding with glucose alone), whereas carbons from mannose are indeed fuelled towards biofuel precursors. In terms of pentose usage, when in a mixture with glucose, the author describes that the fate of the pentose carbons is the pentose phosphate (PP) pathway, with very little assimilation into glycolysis, confirming some previous results from the same author (Aristilde *et al*., 2015). Moreover, in this work, the author provides evidence that assimilated pentose carbons were routed to ribonucleotide synthesis, as previously suggested (Aristilde *et al*., 2015), describing also a bias towards *de novo* synthesis of inosine monophosphate (IMP) over uridine monophosphate (UMP). The study also shows the preference of arabinose over xylose for uptake and metabolism in the PP pathway, as it was already described (Ezeji and Blaschek, 2007), together with a higher rate of *de novo* ribonucleotide biosynthesis in the presence of arabinose. However, when analysing biofuel precursors, results show little contribution of the xylose carbons towards biofuel precursors, and fruitless contribution of the arabinose carbons, which end up in acetate instead of solvents.

In summary, the results presented in this work indicated that there are metabolic hierarchies in *C. acetobutylicum* and that such hierarchies facilitate the selective investment of hemicellulosic pentoses towards ribonucleotide biosynthesis and selective contribution of hemicellulosic hexoses towards biofuel precursors.

With this work, metabolite labelling is presented as a useful way to elucidate the fate of the carbons in a complex sample and a powerful tool to design engineered strains for biofuel production. As mentioned before, the composition of lignocellulosic material varies widely with the origin of the sample, and, for an industrial process with a given material, knowing the fate of the carbons from the different sugars is a precious information to consider when designing tailored‐made bacteria for optimal biofuel production. For example, in the case presented here, for higher butanol production, it would be wise to increase galactose assimilation. Also, decreasing the flux towards acetate and acetone production would ensure a profitable arabinose usage, and decreasing IMP production might direct the pentoses carbons towards biofuel precursors, although this approximation might impair bacterial growth. In any case, and as the author herself pointed out in her conclusions, metabolic labelling should rely on detailed transcriptomics and proteomics analysis, in order to better identify the genes to target in a given pathway and to allow successful design of engineered bacteria for biofuel production.

## Conflict of interest

None declared.
